# Serotonin Syndrome in ICU—A Road Less Traveled

**DOI:** 10.5005/jp-journals-10071-23222

**Published:** 2019-08

**Authors:** Swati Jindal, Satinder Gombar, Kompal Jain

**Affiliations:** 1–3 Department of Anesthesia and Intensive Care, Government College and Hospital, Chandigarh, India

**Keywords:** Agitation, ICU, SSRI, Serotonin syndrome

## Abstract

A potentially life-threatening adverse drug reaction, serotonin syndrome (SS) may occur in patients on serotonin agonist medications. Medications responsible for SS include commonly prescribed antidepressants, anxiolytics, analgesics and antiemetics. This syndrome has been mentioned in various scenarios in perioperative setting but rarely acknowledged in intensive care unit (ICU) setting. Multiple comorbities leading to polypharmacy and serotoninergic medications place elderly patients, particularly at higher risk of SS. We wish to report a case of SS in an elderly male with depression who developed SS and required ICU admission. Most crucial point is to be able to recognize this entity whenever a patient on multiple drugs with altered sensorium is admitted to ICU.

**How to cite this article:**

Jindal S, Gombar S, Jain K. Serotonin Syndrome in ICU—A Road Less Traveled. Indian J Crit Care Med 2019;23(8):376–377.

## INTRODUCTION

Serotinin receptor reuptake inhibitors (SSRIs) are one of the most commonly prescribed antidepressant groups of drugs. Overdose of these medications may lead to SS. Although SS is a well-known entity in perioperative setting, but it is less easily recognized in ICU setting.^1^ Therefore, it is often overlooked. Incidence of SS is underestimated probably due to failure to recognize and indiscriminate SSRI prescription.

## CASE DESCRIPTION

A sixty years old male was admitted to our multidisciplinary ICU with complaints of aggression, altered sensorium, tremors, sweating, clonus in legs (myoclonus) and irrelevant talks. Initially he was managed in medicine emergency where a working diagnosis of gastroenteritis induced hyponatremia with sepsis was made. The patient received infusion of 3% NaCl, antibiotics and oxygen supplementation @ 6 L/min through venturi mask. In view of aggression and altered sensorium, CT brain was done, which was normal. An assessment by neurologist was done, who advised for MRI Brain for further evaluation. Patient was kept nil per oral in view of possibility of further deterioration and need for mechanical ventilation. Since there was no improvement, patient was shifted to ICU. In ICU, the intensivist took a detailed history from the attendants of the patient. His medical history included long standing hypertension, diabetes mellitus type 2, coronary artery disease with PTCA for triple vessel disease and a major depressive disorder. Relatives denied any allergies or illicit drug abuse. Patient was taking metoprolol 25 mg once daily, olmesartan 10 mg twice daily, ecospirin 75 mg once daily, atorvastatin 10 mg at night, metformin 1 g twice daily twice daily, pioglitazone 50 mg once daily, regular insulin 16 U and 20 U subcutaneous, pantoprazole 40 mg once daily along with some herbal powder for his comorbidities for the past 10 years. For last two years, patient's depression was controlled with escitalopram (SSRI) 10 mg and clonazepam (benzodiazepine) 0.5 mg. The patient had an acute episode of anxiety five days before admission to ICU, for which psychiatrist had prescribed another SSRI paroxetine 20 mg and alprazolam 0.5 mg. Symptoms did not subside, rather patient developed restlessness in his legs with current like sensation along with loose motions. His symptoms worsened over next 2 days, and he presented to the medical emergency with severe agitation and presenting complaints as described above.

In the ICU, patient was hemodynamically stable but severely agitated and restless and not responding to commands. Laboratory parameters measured included Hb 12.8 g%, TLC 10800/mm^3^, platelet count 2.7 lakh/mm^3^, INR 1.0, Na^+^ 117meq/L, K^+^ 4.5meq/L, serum creatinine 0.9 mg/dL and ABG pH 7.424/PaO^2^ 109/PaCO^2^ 28.9/HCO^3^ 18.6/BD −4.3/SpO^2^ 99%/lactate 0.7/Na^+^ 117/K^+^ 3.9/iCa^+2^ 3.55/glucose 90 mg/dL/Cl^−^ 83 mEq/L. In view of the recent escalation in the doses and addition of other antidepressants (SSRI) and benzodiazepines with concomitant intake of drugs like metoprolol, pantoprazole and some herbal medications associated with typical features, presumptive diagnosis of SS was made as per Hunters criteria. All antidepressants were stopped. Sedation using titrated doses of propofol (10 mg/hr) to maintain Ramsay score of 2–3 was started to alleviate the symptoms and allow the complete excretion of drugs. Patient received oxygen by face mask and was monitored continuously. Over the next 24 hours his agitation subsided and patient was awake and started responding to commands. Later propofol sedation was gradually tapered off. Thereafter, patient was discharged from ICU and referred back to psychiatrist for further management.

## DISCUSSION

SS is a predictable consequence of excessive serotonin agonism at synaptic and peripheral receptors. It may occur either due to intentional or accidental overdose with a single serotonergic agent but, often occurs due to synergistic effect of two or more proserotonergic drugs. SS represents a diagnosis of exclusion as its recognition depends on clinical perception since laboratory scrutiny and imaging do not provide the definitive message.^2^ Of the three sets of criteria to diagnose SS, i.e Sternbach, Hunter and the Radomski, Hunter serotonin toxicity criteria is considered the most accurate with a sensitivity of 84% and specificity of 97%.^3^ Spectrum of symptoms in SS is diverse ranging from tremors and diarrhea which may often be overlooked, to severe neuromuscular rigidity and hyperthermia which can unintentionally further incite administration of pro serotonergic drugs leading to major adverse crisis. Management involves taking proper history with respect to drugs given on prescription, over the counter, illicit and dietary supplements. One has to be aware of serotonergic effects of few frequently administered drugs. Coadministration of antidepressants along with opioids and antiemetics are known to precipitate SS in hospital settings.^4^ Once the offending agent is found, resolution requires cessation of all serotonergic agents. Maintenance of vital parameters and urine output is essential. In agitated patients, sedation with benzodiazepines and propofol or in severe cases muscle paralysis is recommended. Although our patient did not have hyperthermia, but if it occurs, it does not respond to antipyretics because muscle hypermetabolism and not central nervous system effects are responsible for this syndrome. Antihistaminic with serotonin receptor antagonist properties make cyproheptadine an important drug to lessen the symptoms.^5^ Although SS is well recognized entity in perioperative setting, there are very few reports of patients presenting with SS in ICU setting.^6,7^ To conclude, the present case highlights the importance of detailed history taking with respect to drug intake in patients presenting with severe agitation.
